# Electroacupuncture Alleviates Diabetic Neuropathic Pain and Downregulates p-PKC and TRPV1 in Dorsal Root Ganglions and Spinal Cord Dorsal Horn

**DOI:** 10.1155/2023/3333563

**Published:** 2023-02-03

**Authors:** Yi-qi Ma, Qun-qi Hu, Yu rong Kang, Li-qian Ma, Si-ying Qu, Han-zhi Wang, Yin-mu Zheng, Si-yi Li, Xiao-mei Shao, Xiao-yu Li, Han-tong Hu, Yong-liang Jiang, Jian-qiao Fang, Xiao-fen He

**Affiliations:** Key Laboratory of Acupuncture and Neurology of Zhejiang Province, Department of Neurobiology and Acupuncture Research, The Third Clinical Medical College, Zhejiang Chinese Medical University, Hangzhou, Zhejiang 310053, China

## Abstract

Diabetic neuropathic pain (DNP) is a common complication of diabetes. Streptozotocin (STZ)-induced changes of protein in dorsal root ganglion (DRG) and spinal cord dorsal horn (SCDH) are critical for DNP genesis. However, which proteins change remains elusive. Here, the DNP model was established by a single intraperitoneal injection of STZ, accompanied by increased fasting blood glucose (FBG), decreased body weight (BW), and decreased paw withdrawal latency (PWL). Proteins change in L4-L6 DRGs and SCDH of rats were detected. Western blot and immunofluorescence results showed that expression levels of phosphorylated protein kinase C (p-PKC), transient receptor potential vanilloid-1 (TRPV1), Substance P (SP) and calcitonin gene-related peptide (CGRP) in the DRG and the SCDH of rats were increased after STZ injection. A preliminary study from our previous study showed that 2 Hz electroacupuncture (EA) effectively alleviates DNP. However, the analgesic mechanism of EA needs further elucidation. Here, EA at the bilateral Zusanli (ST36) and KunLun (BL60) acupoints was applied for one week, and to investigate the effect on DNP. EA reversed thermal hyperalgesia in DNP rats and downregulated the expression of p-PKC, TRPV1, SP, and CGRP in DRG and SCDH.

## 1. Introduction

Diabetes is a common metabolic disease [1], and the incidence of diabetes is on the rise [2]. Hyperglycemia can induce metabolic, microvascular lesions, and cause various acute and chronic neuropathy conditions [3]. Diabetic neuropathic pain (DNP) is a major complication of diabetes [4–6], which is mainly characterized by spontaneous pain, paresthesia and hyperalgesia, leading to a decrease in the quality of life of patients [7–9]. The mechanisms underlying DNP still remain unclear, and need further elucidation to produce the effectiveness of some conventional treatment options for DNP.

Dorsal root ganglion (DRG) neurons are the primary afferent nerve cells for trunk and extremity nociception. DRGs are implicated in transmitting and accommodating sensations and receiving and communicating nociception, and they play an important role in the mechanism of pain. Pain signals are transmitted from DRGs to the spinal cord dorsal horn (SCDH) [10, 11]. Neurons in the central processes of the horn and neurons in the DRG form the primary synapse, in which SCDH plays a role in relaying and processing sensory information. Therefore, DRGs and SCDH are key sites for studying neuropathic pain mechanisms. Previous studies report that several DRG pain-related ion channels, receptors and neuropeptides such as Ca^2+^ channels, Na^+^ channels, phosphorylated protein kinase C (p-PKC), transient receptor potential vanilloid-1 (TRPV1) [12], calcitonin gene-related peptide (CGRP), and substance P (SP) [13] are implicated in the transmission of pain. Preliminary studies indicate that p-PKC, TRPV1, SP, and CGRP in DRG play fundamental roles in acute neurogenic inflammation [12]. However, the changes in p-PKC, TRPV1, SP, and CGRP expression in DRG and SCDH in DNP model have not been systematically studied.

Although clinical drugs are used to alleviate DNP, clinical studies have failed to prove the effectiveness of treatment with less adverse effects [14, 15]. Electroacupuncture (EA) therapy is an effective option for chronic pain, including DNP treatment [16], which combines electrical stimulation with the use of acupuncture needles [17–19]. Our previous study showed that 2 Hz EA was more effective than 100 Hz EA in relieving DNP [20]. However, the precise mechanism of 2 Hz EA on DNP has not been fully elucidated.

The present study sought to explore the effect of STZ administration on expressions of p-PKC, TRPV1, SP, and CGRP in DRG and SCDH. These findings will provide a basis for understanding the mechanism of DNP. Moreover, the effect of 2 Hz EA treatment on the expression levels of p-PKC, TRPV1, SP, and CGRP in DRGs and SCDH of DNP rats was explored.

## 2. Materials and Methods

### 2.1. Animals

Male Sprague-Dawley rats (180 ± 20 g) were used in the present study. Rats were assigned to five groups and lived in separate cages. Animals had free access to food and water. Rats were maintained in a controlled environment (20–24°C and 40–60%) with 12-h light/dark cycles at the Animal Laboratory Center of Zhejiang Chinese Medical University (SYXK (zhe) 2018-0012). Experiments were conducted after acclimatization of animals for a week. All experimental procedures were conducted according to animal management regulations. The Animal Welfare Committee of Zhejiang Chinese Medical University approved all protocols in the present study (IACUC-20190805-04).

### 2.2. Establishment of the DNP Rat Model

Rats were fasted for 16 hours and STZ (65 mg/kg, S0130, Sigma) dissolved in sodium citrate buffer (0.1 mol/L, pH 4.5) was administered into rats intraperitoneally [21, 22]. Rats in the Control group received the same volume of the vehicle. Fasting blood glucose (FBG) was determined 3 days after STZ injection. Rats with FBG >13.9 mmol/L [23, 24] and thermal nociceptive sensitivity were used as the criteria for a DNP rat model. Animals that met these criteria were used in subsequent experiments.

### 2.3. Experimental Procedures

The experiment was split into two phases. The effect of STZ on inducing diabetic neuropathic pain was evaluated in the first phase. Rats were randomly assigned to two groups: (1) Control group (*n* = 10, all rats were sacrificed and tissues were harvested after 3 weeks of experiment); (2) STZ group (*n* = 30, 10 rats were killed and tissues were harvested after 1 week, 2 weeks and 3 weeks of experiment). Expression levels of p-PKC, TRPV1, SP, and CGRP in lumbar 4–6 SCDH and DRGs were determined by western blot (WB) or immunofluorescence (IF) analysis. In the second phase, the analgesic effect of EA on DNP and whether p-PKC, TRPV1, SP, and CGRP are implicated in this effect was explored. Rats were randomly assigned to three groups (*n* = 8): (1) Control group; (2) STZ group; and (3) STZ + EA group. Rats in the STZ + EA group were administered with EA daily for a week from the 2 weeks. Tissues were harvested after treatment for western blot and immunofluorescence analysis. Expression levels of p-PKC, TRPV1, SP, and CGRP in lumbar 4–6 SCDH and DRGs were determined by WB or IF.

### 2.4. Determination of Fasting Blood Glucose and Body Weight

Rats were fasted for 8 h and weighed. Blood was obtained from the tail and analysis of FBG was performed using ACCU-CHEK Performa blood glucose meter (Roche Diagnostics GmbH, Germany) a day before administration of STZ and 1 week, 2 weeks, and 3 weeks after STZ injection.

### 2.5. Assessment of Thermal Hypersensitivity

Paw withdrawal latency (PWL) analysis was conducted using the plantar test (37370, Ugo Basile, Italy). Rats were acclimatized in the Plexiglas cubicles (11.5 cm × 17 cm × 14 cm) on the glass plate for at least 30 min, before evaluation. The cut-off time was set at 30 s, and the radiant heat was set to 40, to avoid damage of rat tissue. The light beam was turned off and the timing stopped when the rat raised its paw. The experiment was conducted 3 times per rat with an interval of 5 min between replicates. PWL was calculated as the average of the latencies in seconds.

### 2.6. EA Treatment

Rats in STZ + EA group received EA treatment once a day for one week. Rats that received EA were not anesthetized, but immobilized gently with a self-made retainer. The selected acupoints were bilateral Zusanli (ST36, 5 mm below the fibular head and 1 mm outside the anterior tibial edge) and Kunlun (BL60, depression between the lateral ankle joint and achilles tendon of the hind limb) points. The acupuncture needles (0.25 mm × 13 mm, Hua Tuo, Suzhou Medical Appliance Factory, Jiangsu Province) were carefully inserted into the acupuncture points, and then the acupuncture needles were inserted at a depth of 3 mm for the Kunlun point and 7 mm for the Zusanli point, and then connected to the HANS acupoint electrical stimulation device (Hans-200A, Jisheng Medical Technology, Beijing, China) for 30 minutes. The HANS acupoint electrical stimulation device was set at 1 mA and 2 Hz. Rats in the other groups underwent the same sedation process without EA stimulation.

### 2.7. Western Blot

Experimental rats were anesthetized with sodium pentobarbital (80 mg/kg, i.p), then SCDH and L4-L6 DRGs were harvested. The tissues were homogenized in RIPA Lysis Buffer (P0013B, Beyotime, China) containing a mixture of protease inhibitors (P1050, Beyotime, China) and phosphatase inhibitors (P1050, Beyotime, China) and then centrifuged at 12000 × rpm at 4°C for 20 min. The supernatant was used to identify protein concentration using BCA Protein Assay Kits (23225, Thermo Fisher, USA). The supernatant was diluted with 2 × loading buffer solution and boiled at 100°C for 3 min. Equal amounts of proteins (20 *μ*g) were separated using SDS-PAGE gels electrophoresis and transferred to polyvinylidene difluoride membranes. Subsequently, the membranes were incubated in 5% nonfat milk diluted with 1 × TBST (pH 7.5) for 1 h. Further, the membranes were incubated with rabbit anti-phospho-PKC (1 : 1000; AF3197, Affinity, USA), rabbit anti-TRPV1 (1 : 1000; ACC030, Alomone, USA), and *β*-actin (1 : 5000; #12262, Cell Signaling Technology, USA) overnight at 4°C. Membranes were washed three times with 1 × TBST, 10 min and incubated with HRP-linked antibody (1 : 5000; #7074, Cell Signaling Technology, USA) for 2 h at room temperature. The membranes were then visualized by chemiluminescence (ECL Plus; Beyotime, China), and proteins bands were quantified using the Image Quant LAS 4000 system. Target protein levels were normalized against *β*-actin expression levels.

### 2.8. Immunofluorescence Analysis

Rats were anesthetized with sodium pentobarbital (80 mg/kg, i.p) and transcardially perfused with 4°C saline followed by 4% paraformaldehyde. The spinal cord and DRGs from L4 to L6 were harvested, postfixed in 4% paraformaldehyde for 4 h, and then dehydrated in 15% sucrose solution for 24 h and 30% for 48 h. Tissue sections were prepared using a frozen microtome (30 *μ*m thickness for the spinal cord and 10 *μ*m thickness for DRGs) and subsequently fixed onto glass slides. Sections were rinsed thrice with 1 × TBST for 10 min for each rinse, then blocked with 10% donkey serum for 1 h at 37°C. Sections were incubated with diluted guinea pig anti-TRPV1 (1 : 200; ACC-030-GP, Alomone, Israel) antibodies mixed with rabbit anti-SP (1 : 1500; ab67006, Abcam, UK) or rabbit anti-CGRP (1 : 800; #14959, Cell Signaling Technology) antibodies overnight at 4°C. Tissues slices were washed 6 times in 1 × TBST, for 10 min per wash, then incubated with Goat Anti-Guinea pig IgG H&L (ALexa Fluor® 488) (1 : 600; ab150185, Abcam, UK) and Goat Anti-Rabbit IgG H&L (Alexa Fluor® 594) (1 : 800; ab150084, Abcam, UK) for 1 h at 37°C. Tissue sections were sealed with antifade solution. The sections were then imaged under an Imager M2 microscope (ZEISS, Germany). The scale bar for SCDH slices was 100 *μ*m and the objective magnification was 10×. The scale bar for DRG slices was 50 *μ*m and the objective magnification was 20×. The mean fluorescence intensity of SP and CGRP in SCDH was determined by Image J and the number of SP, CGRP, and TRPV1 positive cells in DRGs was evaluated. Three sections were selected for each rat and three rats were analyzed for each group.

### 2.9. Statistical Analysis

Statistical analysis was conducted using SPSS 22.0 software. Data were presented as mean ± standard error of the mean (SEM). Independent *t*-test was carried out to compare two groups and one-way ANOVA followed by LSD or Dunnett's post hoc tests were used for the comparison of three or more groups. *P* < 0.05 was considered statistically significant.

## 3. Results

### 3.1. Thermal Hyperalgesia in a Rat Model of STZ-Induced Diabetes

**Figure 1:**
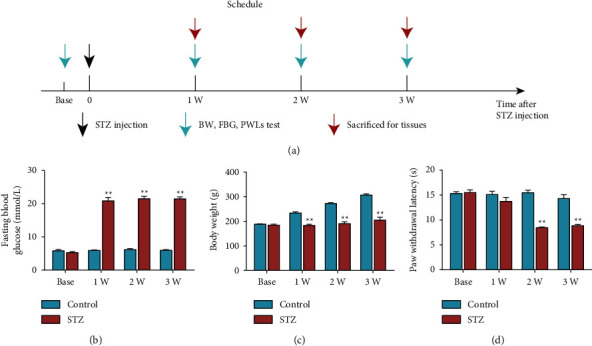
Establishment of DNP rat model by STZ administration. (a) Procedure for generating the DNP rat model. Time course effect of STZ on FBG (b), BW (c), and PWL (d). Data are presented as mean ± SEM, *n* = 10 per group. ^*∗∗*^*P* < 0.01 vs. Control group.

The experimental design for the first phase is given in Figure 1(a). The FBG in the STZ group was higher than the Control group on 1, 2, and 3 weeks (Figure 1(b), *P* < 0.01, respectively). The BW in the STZ group was lower than the Control group on 1, 2, and 3 weeks (Figure 1(c), *P* < 0.01, respectively). The PWL in the STZ group was lower than the Control group on 2 and 3 weeks (Figure 1(d), *P* < 0.01, respectively). These results revealed that the DNP model was successfully established on day 14 after STZ injection.

### 3.2. p-PKC, TRPV1, SP, and CGRP are Increased in the DRG after STZ Injection

**Figure 2:**
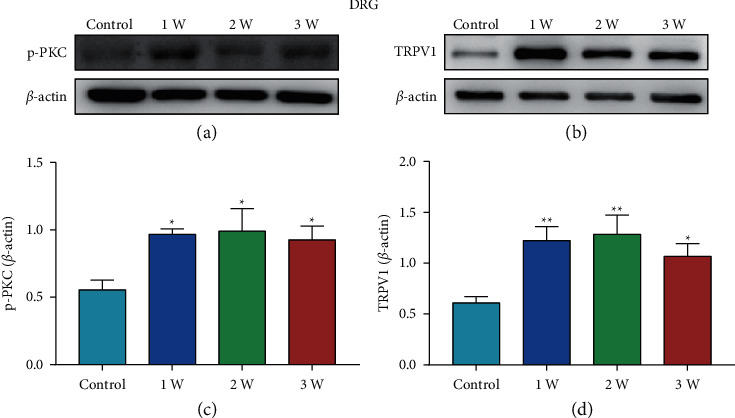
Protein expression of p-PKC and TRPV1 in DRG of rats in STZ group. (a, b) Representative images of WB result of p-PKC and TRPV1 in DRG from different groups. (c, d) WB showed the increased p-PKC and TRPV1 expression in DRG in STZ group rats compared to control rats. Data are presented as mean ± SEM, *n* = 5 per group. ^*∗*^*P* < 0.05, ^*∗∗*^*P* < 0.01 vs. Control group.

**Figure 3:**
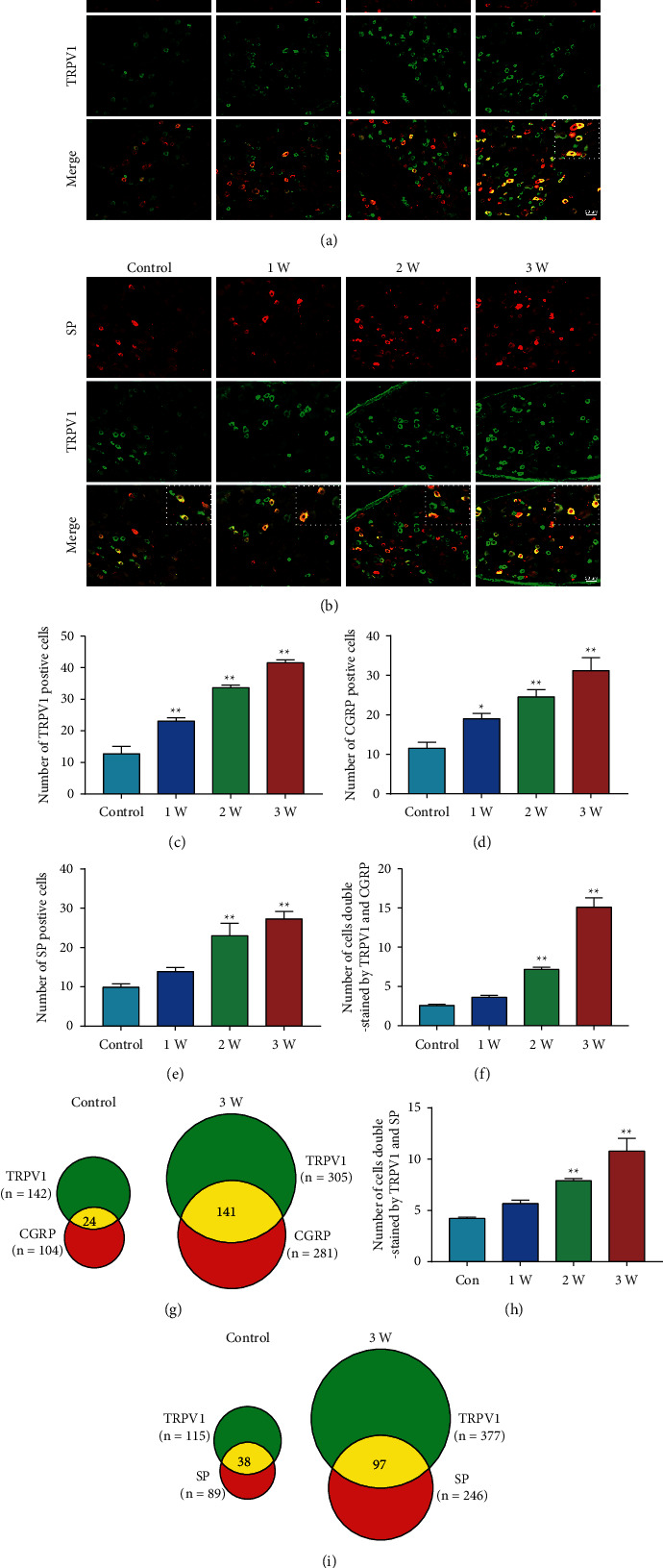
IF results of TRPV1, SP, and CGRP in DRG of rats in STZ group. (a) Representative images of IF of CGRP (red) and TRPV1 (green) in the DRGs from different groups. Scale bar: 50 *μ*m. (b) Representative images of IF of SP (red) and TRPV1 (green) in DRG from different groups. Scale bar: 50 *μ*m. (c) Number of TRPV1 positive cells in DRG from different groups. (d) Number of CGRP positive cells in DRG from different groups. (e) Number of SP positive cells in DRG from different groups. (f) Number of cells double stained by TRPV1 and CGRP in DRG from different groups. (g) The Venn diagram shows the number of neurons double-stained by TRPV1 and CGRP in L4-L6 DRGs, *n* = 3 rats. (h) Number of cells double stained by TRPV1 and SP in DRG from different groups. (i) The Venn diagram shows the number of neurons double-stained by TRPV1 and SP in L4-L6 DRGs. *n* = 3 rats. Data are presented as mean ± SEM, *n* = 3 per group. ^*∗*^*P* < 0.05, ^*∗∗*^*P* < 0.01 vs. Control group.

To investigate the effect of STZ injection on the expression of p-PKC, TRPV1, SP, and CGRP in the L4-L6 DRGs, we used WB and IF to measure those protein levels. WB results showed that STZ injection significantly increased the expressions of p-PKC, TRPV1, SP, and CGRP in the L4-L6 DRGs on 1 W, 2 W, and 3 W (Figure 2(c), *P* < 0.05, *P* < 0.05, *P* < 0.05; Figure 2(d), *P* < 0.01, *P* < 0.01, *P* < 0.05). Double immunofluorescence assays were performed to explore whether SP/TRPV1 and CGRP/TRPV1 were coexpressed in DRG cells (Figure 3). SP is a peptide mainly secreted by neurons and is involved in neurotransmission during injuries [25]. Moreover, CGRP is implicated in the transmission of pain signals [26]. The findings showed that CGRP was coexpressed with TRPV1 in DRG cells (Figure 3(a)), and SP was coexpressed with TRPV1 in DRG cells (Figure 3(b)). In addition, positive cell counts showed that the number of TRPV1-positive, CGRP-positive in DRG was increased significantly starting 1 week after STZ injection (Figure 3(c), *P* < 0.01, *P* < 0.01, *P* < 0.01; Figure 3(d), *P* < 0.05, *P* < 0.01, *P* < 0.01). SP-positive, TRPV1/CGRP-positive, and TRPV1/SP-positive cells in DRG were significantly increased starting 2 week after STZ injection (Figure 3(e), *P* < 0.01, *P* < 0.01, Figure 3(f), *P* < 0.01, *P* < 0.01Figure 3(h), *P* < 0.01, *P* < 0.01). Venn diagram showed that the number of coexpressing cells in the DRG of the 3 W group was significantly increased compared to Control group (Figures 3(g) and 3(i)).

### 3.3. p-PKC, TRPV1, SP, and CGRP are Increased in the SCDH after STZ Injection

**Figure 4:**
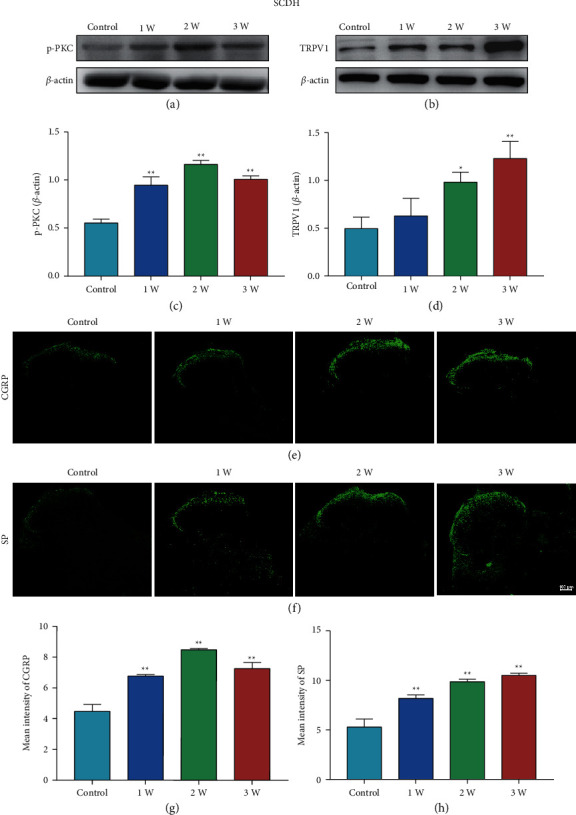
Protein expression of p-PKC and TRPV1 in SCDH of rats in STZ group. (a, b) Representative images of WB result of p-PKC and TRPV1 in SCDH from different groups. (c, d) WB showed the increased p-PKC and TRPV1 expression in SCDH in STZ group rats compared to Control rats. Data are presented as mean ± SEM, *n* = 5 per group. (e) Representative images of CGRP staining in SCDH. (f) Representative images of SP staining in SCDH. (g) Mean intensity analysis of CGRP staining in SCDH. (h) Mean intensity analysis of SP staining in SCDH. Scale bars=100 μm. Data are presented as mean ± SEM, *n* = 3 per group. ^∗^*P* < 0.05, ^∗∗^*P* < 0.01 vs. Control group.

**Figure 5:**
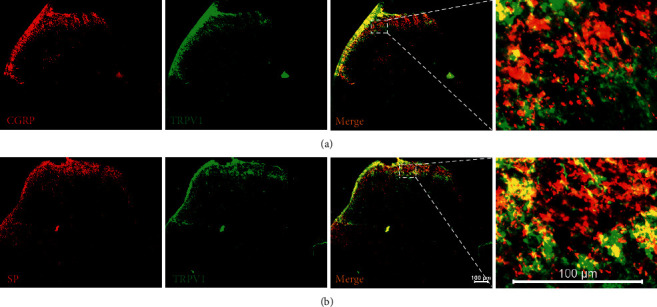
(a) Representative images of IF of CGRP/TRPV1 coexpression in SCDH. (b) Representative images of IF of SP/TRPV1 coexpression in SCDH Scale bars=100 μm. Data are presented as mean ± SEM, *n* = 3 per group.

The expression levels p-PKC, TRPV1, SP, and CGRP in SCDH were determined to explore the effect of STZ on SCDH (Figures 4 and 5). WB results indicated that the p-PKC protein was increased from one to three weeks (Figure 4(c), *P* < 0.01, *P* < 0.01, *P* < 0.01), and TRPV1 protein was increased from two to three weeks (Figure 4(d), *P* < 0.05, *P* < 0.01). IF results showed SP and CGRP increased from one to three weeks (Figures 4(g) and 4(h), *P* < 0.01, respectively). Moreover, IF results demonstrated the coexpression of SP/TRPV1 and CGRP/TRPV1 in the SCDH (Figures 5(a) and 5(b)).

### 3.4. EA Alleviates Thermal Hyperalgesia in a Rat Model of STZ-Induced DNP

**Figure 6:**
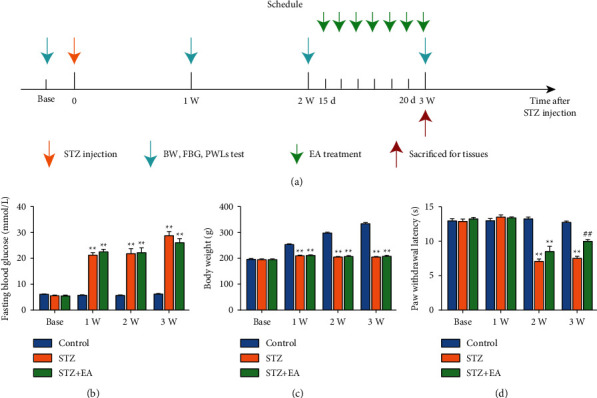
EA attenuated thermal hyperalgesia in a rat model of DNP. (a) Experimental design for establishment of DNP rat model and EA treatment. Time course effect of STZ and EA treatment on FBG (b), BW (c) and PWL (d). Data are presented as mean ± SEM, *n* = 10 per group. ^∗∗^*P* < 0.01 vs. Control group; ^##^*P* < 0.01 vs. STZ group.

The experimental design is given in Figure 6(a). The FBG in the STZ group was increased, and the BW in the STZ group was decreased at 1, 2, and 3 weeks (Figures 6(b) and 6(c), *P* < 0.01, respectively). The PWL in the STZ group decreased at 2 and 3 weeks, indicating the successful establishment of DNP in rats (Figure 6(d), *P* < 0.01, respectively). The rats in the STZ + EA group were treated with EA from the 15th day to the 21st day. EA reduced STZ-induced thermal hyperalgesia in DNP rat models in the third week (Figure 6(d), *P* < 0.01). However, EA did not produce an effect on FBG and BW in DNP rats in the third week (Figures 6(b) and 6(c), *P* > 0.05, respectively).

### 3.5. EA Reduces Expression of p-PKC, TRPV1, SP, and CGRP in the DRG of DNP Rats

**Figure 7:**
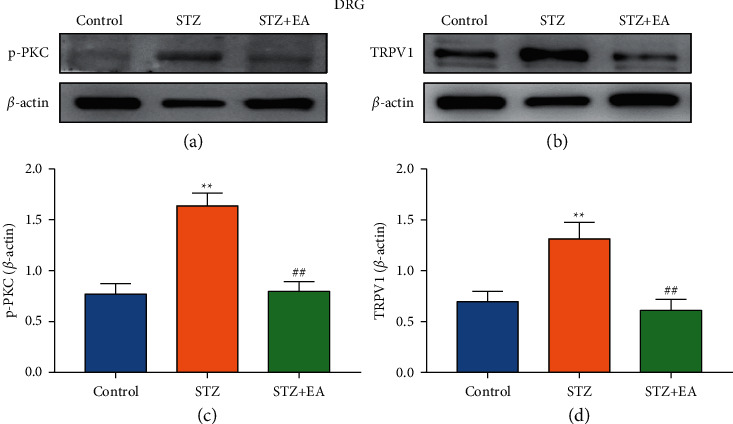
Protein expression of p-PKC and TRPV1 in DRG of DNP rats after EA treatment. (a, b) Representative images of WB result of p-PKC and TRPV1 in DRG from different groups. (c, d) WB showed the decreased p-PKC and TRPV1 expression in DRG in STZ + EA group rats compared to DNP rats. Data are presented as mean ± SEM, *n* = 5 per group. ^*∗*^*P* < 0.05, ^*∗∗*^*P* < 0.01 vs. Control group; ^#^*P* < 0.05, ^##^*P* < 0.01 vs. STZ group.

**Figure 8:**
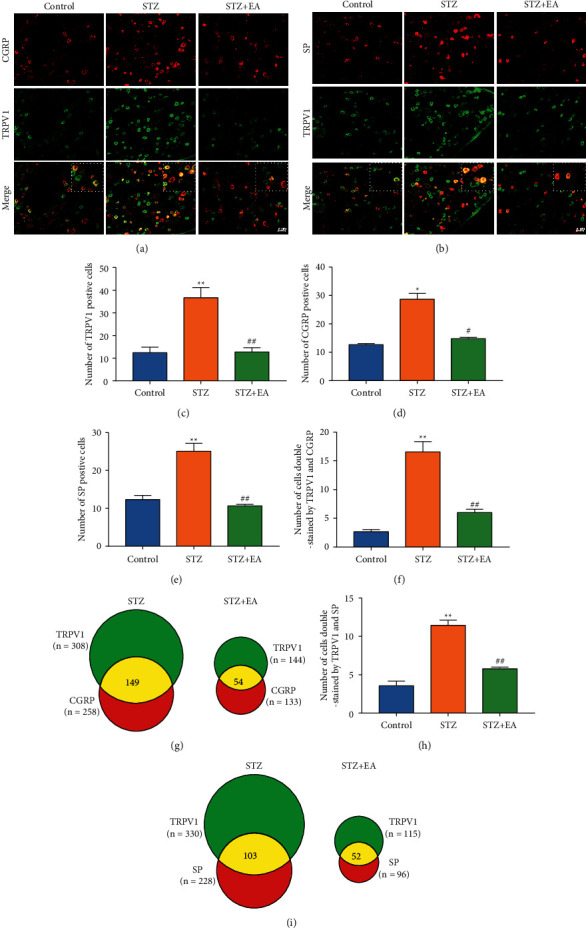
IF results of TRPV1, SP, and CGRP in DRG of DNP rats after EA treatment. (a) Representative images of IF of CGRP (red) and TRPV1 (green) in DRG from different groups. Scale bar: 50 *μ*m. (b) Representative images of IF of SP (red) and TRPV1 (green) in DRG from different groups. Scale bar: 50 *μ*m. (c) Number of TRPV1 positive cells in DRG from different groups. (d) Number of CGRP positive cells in DRG from different groups. (e) Number of SP positive cells in DRG from different groups. (f) Number of cells double stained by TRPV1 and CGRP in DRG from different groups. (g) The Venn diagram shows the number of cells double stained with TRPV1 and CGRP in rat L4-L6 DRGs from various groups. *n* = 3 rats. (h) Number of cells double stained by TRPV1 and SP in DRG from different groups. (i) The Venn diagram shows the number of cells double stained with TRPV1 and SP in rat L4-L6 DRGs from various groups. *n* = 3. Scale bars = 50 *μ*m. Data are presented as mean ± SEM, *n* = 3 per group. ^*∗*^*P* < 0.05, ^*∗∗*^*P* < 0.01 vs. Control group; ^#^*P* < 0.05, ^##^*P* < 0.01 vs. STZ group.

Further WB and IF analyses were conducted to explore the effect of EA treatment on p-PKC, TRPV1, SP, and CGRP expression levels in L4-L6 DRGs of DNP rats (Figure 7). WB analysis showed p-PKC and TRPV1 expression in L4-L6 DRGs increased remarkably, compared to that of the Control rats (Figures 7(c) and 7(d), *P* < 0.01). EA treatment downregulated p-PKC and TRPV1 expression (Figures 7(c) and 7(d), *P* < 0.01). IF analysis showed that the numbers of TRPV1-positive, CGRP-positive, SP-positive, TRPV1/CGRP-positive, and TRPV1/SP-positive cells in DRG were significantly upregulated (Figures 8(c)–8(f) and 8(h), *P* < 0.01, *P* < 0.05, *P* < 0.01, *P* < 0.01, *P* < 0.01). EA treatment remarkably attenuated the upregulated number of those positively cells (Figures 8(c)–8(f) and 8(h), *P* < 0.01, *P* < 0.05, *P* < 0.01, *P* < 0.01, *P* < 0.01). The Venn diagram showed that the number of coexpressing cells in the DRG of the STZ + EA group was significantly lower than that of the STZ group (Figures 8(g) and 8(i)).

### 3.6. EA Reduces Expression of p-PKC, TRPV1, SP, and CGRP in the SCDH of DNP Rats

**Figure 9:**
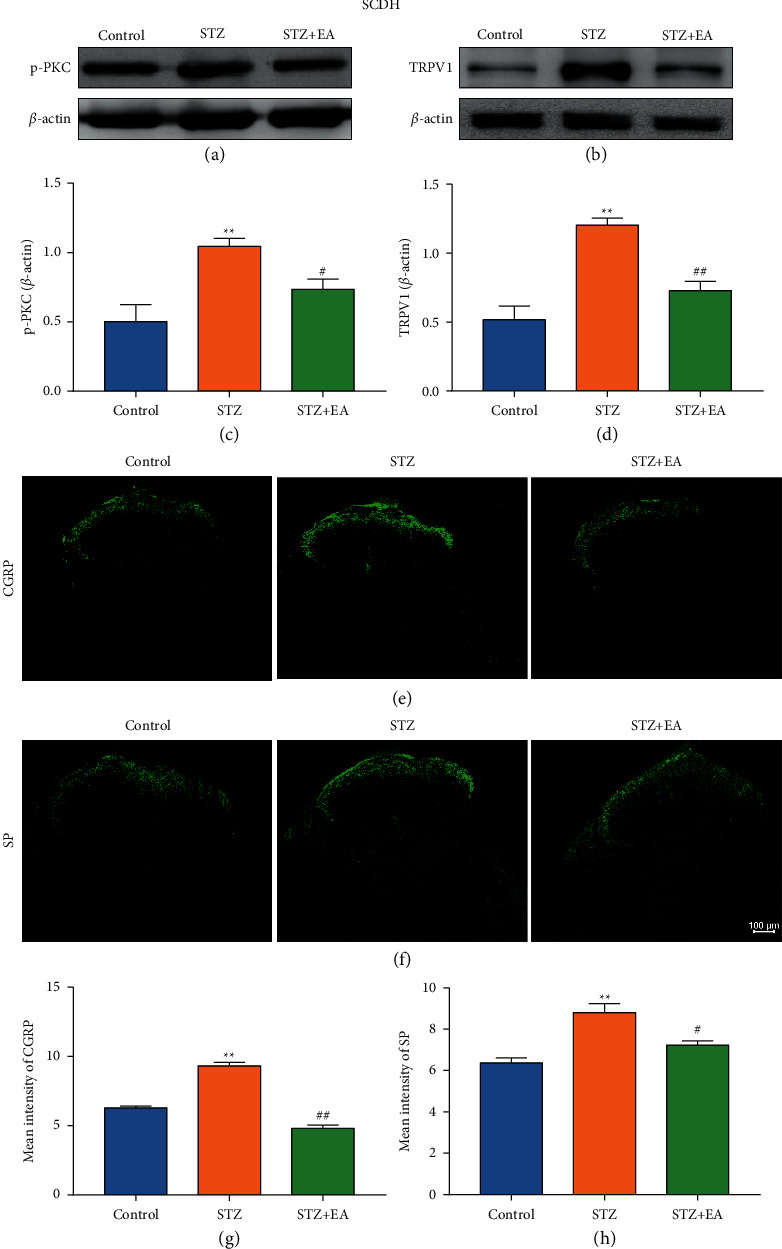
Expression of p-PKC and TRPV1 in SCDH of DNP rats after EA treatment. (a, b) Representative images of WB result of p-PKC and TRPV1 in SCDH from different groups. (c, d) WB showed the decreased p-PKC and TRPV1 expression in SCDH in STZ + EA group rats compared to DNP rats. Data are presented as mean ± SEM, *n* = 5 per group. (e) Representative images of IF of CGRP staining in SCDH from different groups. (f) Representative images of IF of SP staining in SCDH from different groups. (g) Mean intensity analysis of CGRP in SCDH from various groups. (h) Mean intensity analysis of SP in SCDH from different groups. Scale bars = 100 *μ*m. Data are presented as mean ± SEM, *n* = 3 per group. ^*∗∗*^*P* < 0.01 vs. Control group; ^#^*P* < 0.05, ^##^*P* < 0.01 vs. STZ group.

Further WB and IF analyses were conducted to explore expression levels of p-PKC, TRPV1, SP, and CGRP in SCDH of DNP rats after EA treatment (Figure 9). WB results indicated that p-PKC and TRPV1 expression increased remarkably. EA treatment decreased the increased expressions of p-PKC and TRPV1 (Figure 9(c), *P* < 0.01, *P* < 0.05; Figure 9(d), *P* < 0.01, *P* < 0.01). IF results indicated that STZ injection significantly increased the mean intensity of SP and CGRP in L4-6 SCDH (Figures 9(g) and 9(h), *P* < 0.01). Notably, EA stimulation remarkably reduced the mean intensity of SP and CGRP in L4-6 SCDH (Figure 9(g), *P* < 0.01; Figure 9(h), *P* < 0.05).

## 4. Discussion

In the current study, we investigated the changes of p-PKC, TRPV1, SP, and CGRP protein in DRG and SCDH in STZ-induced neuropathic pain. The results showed that the expressions of p-PKC, TRPV1, SP, and CGRP were increased in L4-6 DRG and SCDH, and TRPV1 was coexpressed with SP, and TRPV1 was also coexpressed with CGRP. We then examined the effect of 2 Hz EA on the thermal hyperalgesia of DNP model rats. In total, 2 Hz frequency of EA was applied for 30 minutes every day after DNP model establishment, from days 15 to 21. Results indicated that 2 Hz EA produced antiallodynic effect on DNP model rats, and EA effectively reduced overexpression of the p-PKC, TRPV1, SP, and CGRP marker proteins.

STZ is a glucosamine-nitrosourea that can selectively destroy pancreatic islet *β*-cells in mammals [27] and is commonly used in establishing diabetes models [28]. In this study, FBG increased and BW decreased remarkably starting at 1 week after STZ injection. PWL decreased remarkably starting at 2 weeks after STZ injection, indicating the successful establishment of the DNP model, which consisted with our previous research [29].

DRGs and SCDH play vital roles in many neuropathic pain [30–32]. DRG receives pain signals and transmits them to the SCDH [33, 34]. Many changes of protein in DRG and SCDH are involved in neuropathic pain [35–37].

Previous studies showed that PKC is involved in the transmission of neuropathic pain including DNP [14, 38, 39]. PKC is a phospholipid-dependent serine/threonine kinase family. This family comprises 13 isoenzymes that can be activated by extracellular signals [40]. The active state of PKC is p-PKC, which is a phosphorylated state [41, 42] and is implicated in various roles [43]. TRPV1 is a nonselective ligand-gated cationic channel assembled as a homotetramer and widely distributed in SCDH and DRGs [12, 44, 45]. TRPV1 receives various pain-causing stimuli such as noxious heat and diverse chemical irritants or toxins [46–48]. TRPV1 is an effective target for control of neuropathic pain [49]. A previous study reported that the expression of p-PKC and TRPV1 in neurogenic inflammation was significantly upregulated in DRGs [12]. This is consistent with the results of the present study. In the current study, WB analysis showed an increase in p-PKC and TRPV1 expression levels in DRGs and SCDH of DNP rats. SP and CGRP are coexpressed in primary sensory nerves. IF results showed that the number of TRPV1-positive and CGRP-positive in DRG were increased significantly starting 1 week after STZ injection. SP-positive, TRPV1/CGRP-positive, and TRPV1/SP-positive cells in DRG were significantly increased starting 2 weeks after STZ injection. P-PKC, CGRP, and SP in SCDH are significantly elevated starting from the first week, while TRPV1 in SCDH was significantly increased from the 2 weeks. This may be why thermal hyperalgesia developed at 2 weeks rather than 1 week after STZ injection. Sensory nerves endings are released to transmit pain signals when they are activated by stimuli [50]. SP and CGRP are expressed after activation of TRPV1 [51]. In the present study, immunofluorescence double staining was performed to explore colocalization of TRPV1 with SP and CGRP and to verify the upregulation of SP and CGRP expression in DNP. The findings indicated that STZ injection induces expression of p-PKC, TRPV1, SP, and CGRP in DRGs and SCDH upregulated.

Currently, clinical studies have failed to prove the effectiveness of treatment with less adverse effects for patients with neuropathic pain [52]. A previous study reported that berberine blocks PKC channels to inhibit TRPV1 activation, thus improving DNP [14]. EA is a combination of acupuncture and electric current and is an effective approach for relieving neuropathic pain [53]. A previous study reported that 2 Hz EA has better analgesic effects than 100 Hz EA [20]. The analgesic effect of 2 Hz EA has also been demonstrated in other pain models [54, 55]. Numerous studies have shown that EA intervention on ST36 and BL60 in rats can alleviate different types of neuropathic pain [56–58]. The preliminary study of our research group showed that the intervention of EA of ST36 and BL60 can effectively alleviate diabetic neuropathic pain [59, 60]. Thus, in the present study, the acupoints of ST36 and BL60 were selected to study the analgesic mechanism of EA. EA intervention in rats with neck-incision pain upregulated thermal pain thresholds and downregulated CGRP and SP expression in the dorsal aspect of the cervical spinal cord [61]. In addition, EA ameliorated nociceptive sensitization in rats with chronic pain and reduced TRPV1 expression on DRG [56]. EA treatment improved thermal hyperalgesia. EA treatment significantly reduced the overexpression of p-PKC, TRPV1, SP, and CGRP in SCDH and DRGs of DNP rats. These findings all support that EA may be a promising therapeutic option for DNP. However, further clinical studies are needed to comprehensively evaluate the therapeutic potentials of EA on DNP patients.

## 5. Conclusion

In conclusion, p-PKC, TRPV1, SP, and CGRP in DRGs and SCDH were significantly elevated after STZ-induced neuropathic pain. EA treatment alleviates STZ-induced DNP, which may be associated with downregulation of p-PKC, TRPV1, SP, and CGRP in DRGs and SCDH. However, the specific mechanism of action of EA was not explored in the current study. Further studies should be conducted to determine the role of p-PKC/TRPV1 in DNP.

## Data Availability

Key data are included in the diagrams and the main text. Datasets used and analyzed in this study are available upon request from the corresponding author.

## Abbreviations

BW: Body weight
CGRP: Calcitonin gene-related peptide
DNP: Diabetic neuropathic pain
DRG: Dorsal root ganglion
EA: Electroacupuncture
FBG: Fasting blood glucose
IF: Immunofluorescence
i.p: Intraperitoneal
PKC: Protein kinase C
p-PKC: Phosphorylated protein kinase C
PWL: Paw withdrawal latency
SCDH: Spinal cord dorsal horn
SEM: Standard error of the mean
SP: Substance P
STZ: Streptozotocin
TRPV1: Transient receptor potential vanilloid-1
WB: Western blot.

## References

[1] Veiseh O., Tang B. C., Whitehead K. A., Anderson D. G., Langer R. (2015). Managing diabetes with nanomedicine: challenges and opportunities. *Nature Reviews Drug Discovery*.

[2] An X., Jin D., Duan L. (2020). Direct and indirect therapeutic effect of traditional Chinese medicine as an add-on for non-proliferative diabetic retinopathy: a systematic review and meta-analysis. *Chinese Medicine*.

[3] Tesfaye S., Boulton A. J., Dyck P. J. (2010). Diabetic neuropathies: update on definitions, diagnostic criteria, estimation of severity, and treatments. *Diabetes Care*.

[4] Rosenberger D. C., Blechschmidt V., Timmerman H., Wolff A., Treede R. D. (2020). Challenges of neuropathic pain: focus on diabetic neuropathy. *Journal of Neural Transmission*.

[5] Lu J. H., Zhang M. B., Wang J. W. (2020). Kalirin-7 contributes to type 2 diabetic neuropathic pain via the postsynaptic density-95/N-methyl-D-aspartate receptor 2B-dependent N-methyl-D-aspartate receptor 2B phosphorylation in the spinal cord in rats. *American Journal of Translational Research*.

[6] Tesfaye S., Selvarajah D. (2012). Advances in the epidemiology, pathogenesis and management of diabetic peripheral neuropathy. *Diabetes/Metabolism Research and Reviews*.

[7] Ziegler D., Low P. A., Litchy W. J. (2011). Efficacy and safety of antioxidant treatment with *α*-lipoic acid over 4 years in diabetic polyneuropathy: the nathan 1 trial. *Diabetes Care*.

[8] van Hecke O., Austin S. K., Khan R. A., Smith B. H., Torrance N. (2014). Neuropathic pain in the general population: a systematic review of epidemiological studies. *Pain*.

[9] Van Acker K., Bouhassira D., De Bacquer D. (2009). Prevalence and impact on quality of life of peripheral neuropathy with or without neuropathic pain in type 1 and type 2 diabetic patients attending hospital outpatients clinics. *Diabetes and Metabolism*.

[10] Piedade G. S., Vesper J., Chatzikalfas A., Slotty P. J. (2019). Cervical and high-thoracic dorsal root ganglion stimulation in chronic neuropathic pain. *Neuromodulation: Technology at the Neural Interface*.

[11] Berta T., Qadri Y., Tan P. H., Ji R. R. (2017). Targeting dorsal root ganglia and primary sensory neurons for the treatment of chronic pain. *Expert Opinion on Therapeutic Targets*.

[12] Xu X., Wang P., Zou X., Li D., Fang L., Lin Q. (2009). Increases in transient receptor potential vanilloid-1 mRNA and protein in primary afferent neurons stimulated by protein kinase C and their possible role in neurogenic inflammation. *Journal of Neuroscience Research*.

[13] Lin Y. T., Chen J. C. (2018). Dorsal root ganglia isolation and primary culture to study neurotransmitter release. *Journal of Visualized Experiments*.

[14] Zan Y., Kuai C. X., Qiu Z. X., Huang F. (2017). Berberine ameliorates diabetic neuropathy: TRPV1 modulation by PKC pathway. *American Journal of Chinese Medicine*.

[15] Russell J. W., Zilliox L. A. (2014). Diabetic neuropathies. *Continuum: Lifelong Learning in Neurology*.

[16] Wang X., Li Q., Han X., Gong M., Yu Z., Xu B. (2021). Electroacupuncture alleviates diabetic peripheral neuropathy by regulating glycolipid-related GLO/AGEs/RAGE axis. *Frontiers in Endocrinology*.

[17] Comachio J., Oliveira Magalhães M., Nogueira Burke T. (2015). Efficacy of acupuncture and electroacupuncture in patients with nonspecific low back pain: study protocol for a randomized controlled trial. *Trials*.

[18] Zhang R., Lao L., Berman B. M., Ren K., Berman B. M. (2014). Mechanisms of acupuncture–electroacupuncture on persistent pain. *Anesthesiology*.

[19] Han J. S. (2011). Acupuncture analgesia: areas of consensus and controversy. *Pain*.

[20] He X. F., Wei J. J., Shou S. Y., Fang J. q., Jiang Y. l. (2017). Effects of electroacupuncture at 2 and 100 hz on rat type 2 diabetic neuropathic pain and hyperalgesia-related protein expression in the dorsal root ganglion. *Journal of Zhejiang University—Science B*.

[21] Wu Y., Leng Y., Meng Q. (2017). Suppression of excessive histone deacetylases activity in diabetic hearts attenuates myocardial ischemia/reperfusion injury via mitochondria apoptosis pathway. *Journal of Diabetes Research*.

[22] Feng B., Chen S., McArthur K. (2011). miR-146a–Mediated extracellular matrix protein production in chronic diabetes complications. *Diabetes*.

[23] Du L., Wang L., Wang B. (2020). A novel compound AB38b attenuates oxidative stress and ECM protein accumulation in kidneys of diabetic mice through modulation of Keap1/Nrf2 signaling. *Acta Pharmacologica Sinica*.

[24] Erbaş O., Oltulu F., Yılmaz M., Yavasoglu A., Taskiran D. (2016). Neuroprotective effects of chronic administration of levetiracetam in a rat model of diabetic neuropathy. *Diabetes Research and Clinical Practice*.

[25] Felipe C. D., Herrero J. F., O’Brien J. A. (1998). Altered nociception, analgesia and aggression in mice lacking the receptor for substance P. *Nature*.

[26] Russell F. A., King R., Smillie S. J., Kodji X., Brain S. D. (2014). Calcitonin gene-related peptide: physiology and pathophysiology. *Physiological Reviews*.

[27] Rerup C. C. (1970). Drugs producing diabetes through damage of the insulin secreting cells. *Pharmacological Reviews*.

[28] Ortiz M. d. C., Lores-Arnaiz S., Albertoni Borghese M. F. (2013). Mitochondrial dysfunction in brain cortex mitochondria of STZ-diabetic rats: effect of l-arginine. *Neurochemical Research*.

[29] He X. F., Kang Y. R., Fei X. Y. (2022). Inhibition of phosphorylated calcium/calmodulin-dependent protein kinase II*α* relieves streptozotocin-induced diabetic neuropathic pain through regulation of P2X3 receptor in dorsal root ganglia. *Purinergic Signalling*.

[30] Esposito M. F., Malayil R., Hanes M., Deer T. (2019). Unique characteristics of the dorsal root ganglion as a target for neuromodulation. *Pain Medicine*.

[31] Tsuda M. (2016). Microglia in the spinal cord and neuropathic pain. *Journal of Diabetes Investigation*.

[32] Li J., Wang G., Weng Y., Ding M., Yu W. (2020). Netrin-1 contributes to peripheral nerve injury induced neuropathic pain via regulating phosphatidylinositol 4-kinase IIa in the spinal cord dorsal horn in mice. *Neuroscience Letters*.

[33] Hu J., Huang T., Li T., Guo Z., Cheng L. (2012). c-Maf is required for the development of dorsal horn laminae III/IV neurons and mechanoreceptive DRG axon projections. *Journal of Neuroscience*.

[34] Cheng C. F., Wang W. C., Huang C. Y., Du P. H., Yang J. H., Tsaur M. L. (2016). Coexpression of auxiliary subunits KChIP and DPPL in potassium channel Kv4-positive nociceptors and pain-modulating spinal interneurons. *Journal of Comparative Neurology*.

[35] Zhang Y., Zhao D., Li X. (2021). The wnt/*β*-catenin pathway regulated cytokines for pathological neuropathic pain in chronic compression of dorsal root ganglion model. *Neural Plasticity*.

[36] Zheng X. B., Zhang Y. L., Li Q. (2019). Effects of 1, 8-cineole on neuropathic pain mediated by P2X2 receptor in the spinal cord dorsal horn. *Scientific Reports*.

[37] Yamada M., Fujita Y., Hayano Y. (2019). Increased expression of fibronectin leucine-rich transmembrane protein 3 in the dorsal root ganglion induces neuropathic pain in rats. *Journal of Neuroscience*.

[38] García G., Méndez-Reséndiz K. A., Oviedo N., Murbartian J. (2021). PKC‐ and PKA‐dependent phosphorylation modulates TREK‐1 function in naïve and neuropathic rats. *Journal of Neurochemistry*.

[39] Xie J. D., Chen S. R., Chen H., Pan H. L. (2017). Bortezomib induces neuropathic pain through protein kinase C-mediated activation of presynaptic NMDA receptors in the spinal cord. *Neuropharmacology*.

[40] Talman V., Provenzani R., Boije af Gennäs G., Tuominen R., Yli-Kauhaluoma J. (2014). C1 domain-targeted isophthalates as protein kinase C modulators: structure-based design, structure–activity relationships and biological activities. *Biochemical Society Transactions*.

[41] Nishizuka Y. (1984). The role of protein kinase C in cell surface signal transduction and tumour promotion. *Nature*.

[42] Langham R. G., Kelly D. J., Gow R. M. (2008). Increased renal gene transcription of protein kinase C-*β* in human diabetic nephropathy: relationship to long-term glycaemic control. *Diabetologia*.

[43] Pui Ping C., Akhtar M. N., Israf D. A., Perimal E. K., Sulaiman M. R. (2020). Possible participation of ionotropic glutamate receptors and l-arginine-nitric oxide-cyclic guanosine monophosphate-ATP-sensitive K+ channel pathway in the antinociceptive activity of cardamonin in acute pain animal models. *Molecules*.

[44] Bevan S., Quallo T., Andersson D. A. (2014). Trpv1. *Handbook of Experimental Pharmacology*.

[45] Mandadi S., Tominaga T., Numazaki M. (2006). Increased sensitivity of desensitized TRPV1 by PMA occurs through PKC*ε*-mediated phosphorylation at S800. *Pain*.

[46] Iftinca M., Defaye M., Altier C. (2021). TRPV1-Targeted drugs in development for human pain conditions. *Drugs*.

[47] Cuypers E., Yanagihara A., Karlsson E., Tytgat J. (2006). Jellyfish and other cnidarian envenomations cause pain by affecting TRPV1 channels. *FEBS Letters*.

[48] Hakim M. A., Jiang W., Luo L. (2015). Scorpion toxin, BmP01, induces pain by targeting TRPV1 channel. *Toxins*.

[49] Szallasi A., Cortright D. N., Blum C. A., Eid S. R. (2007). The vanilloid receptor TRPV1: 10 years from channel cloning to antagonist proof-of-concept. *Nature Reviews Drug Discovery*.

[50] Cattaruzza F., Cottrell G., Vaksman N., Bunnett N. (2009). Endothelin-converting enzyme 1 promotes re-sensitization of neurokinin 1 receptor-dependent neurogenic inflammation. *British Journal of Pharmacology*.

[51] Zhang X. Y., Guo Z., Li T. P., Sun T. (2021). Dietary capsaicin normalizes CGRP peptidergic DRG neurons in experimental diabetic peripheral neuropathy. *Scientific Reports*.

[52] Hall S. M., Lee Y. S., Hruby V. J. (2016). Dynorphin A analogs for the treatment of chronic neuropathic pain. *Future Medicinal Chemistry*.

[53] Wei J. A., Hu X., Zhang B. (2021). Electroacupuncture activates inhibitory neural circuits in the somatosensory cortex to relieve neuropathic pain. *iScience*.

[54] Zhao W. S., Jiang Z. N., Shi H., Xu L. l., Yang Y., Wang Y. c. (2019). Low-frequency electroacupuncture alleviates chronic constrictive injury-induced mechanical allodynia by inhibiting NR2B upregulation in ipsilateral spinal dorsal horn in rats. *Chinese Journal of Integrative Medicine*.

[55] Shin K. M., Ko I. G., Kim S. E. (2018). Low-frequency electroacupncture improves locomotor function after sciatic crushed nerve injury in rats. *Journal of Exercise Rehabilitation*.

[56] Liu Y., Du J., Fang J. (2021). Electroacupuncture inhibits the interaction between peripheral TRPV1 and P2X3 in rats with different pathological pain. *Physiological Research*.

[57] Li X., Yin C., Hu Q. (2022). Nrf2 activation mediates antiallodynic effect of electroacupuncture on a rat model of complex regional pain syndrome type-I through reducing local oxidative stress and inflammation. *Oxidative Medicine and Cellular Longevity*.

[58] Yu X., Chen X., Liu W., Jiang M., Wang Z., Tao J. (2021). Proteomics analysis of the spinal dorsal horn in diabetic painful neuropathy rats with electroacupuncture treatment. *Frontiers in Endocrinology*.

[59] Hu Q. Q., He X. F., Ma Y. Q. (2022). Dorsal root ganglia P2X4 and P2X7 receptors contribute to diabetes-induced hyperalgesia and the downregulation of electroacupuncture on P2X4 and P2X7. *Purinergic Signalling*.

[60] Fei X., He X., Tai Z. (2020). Electroacupuncture alleviates diabetic neuropathic pain in rats by suppressing P2X3 receptor expression in dorsal root ganglia. *Purinergic Signalling*.

[61] Qiao L. N., Wang J. Y., Yang Y. S. (2013). Effect of electroacupuncture intervention on expression of cgrp, sp, cox-1, and pge2 of dorsal portion of the cervical spinal cord in rats with neck-incision pain. *Evidence Based Complementary and Alternative Medicine*.

